# Characterization of longitudinal canal tissue in the acorn barnacle *Amphibalanus amphitrite*

**DOI:** 10.1371/journal.pone.0208352

**Published:** 2018-12-10

**Authors:** Chenyue Wang, Janna N. Schultzhaus, Chris R. Taitt, Dagmar H. Leary, Lisa C. Shriver-Lake, Daniel Snellings, Samantha Sturiale, Stella H. North, Beatriz Orihuela, Daniel Rittschof, Kathryn J. Wahl, Christopher M. Spillmann

**Affiliations:** 1 National Research Council Research Associateship Program, Washington, D.C., United States of America; 2 Center for Bio/Molecular Science and Engineering, Naval Research Laboratory, Washington, D.C., United States of America; 3 Naval Research Enterprise Internship Program, Washington, D.C., United States of America; 4 Duke University Marine Laboratory, Beaufort, N.C., United States of America; 5 Chemistry Division, Naval Research Laboratory, Washington, D.C., United States of America; University of Split, Faculty of science, CROATIA

## Abstract

The morphology and composition of tissue located within parietal shell canals of the barnacle *Amphibalanus amphitrite* are described. Longitudinal canal tissue nearly spans the length of side shell plates, terminating near the leading edge of the specimen basis in proximity to female reproductive tissue located throughout the peripheral sub-mantle region, *i*.*e*. mantle parenchyma. Microscopic examination of stained longitudinal canal sections reveal the presence of cell nuclei as well as an abundance of micron-sized spheroids staining positive for basic residues and lipids. Spheroids with the same staining profile are present extensively in ovarioles, particularly within oocytes which are readily identifiable at various developmental stages. Mass spectrometry analysis of longitudinal canal tissue compared to tissue collected from the mantle parenchyma reveals a nearly 50% overlap of the protein profile with the greatest number of sequence matches to vitellogenin, a glycolipoprotein playing a key role in vitellogenesis–yolk formation in developing oocytes. The morphological similarity and proximity to female reproductive tissue, combined with mass spectrometry of the two tissues, provides compelling evidence that one of several possible functions of longitudinal canal tissue is supporting the female reproductive system of *A*. *amphitrite*, thus expanding the understanding of the growth and development of this sessile marine organism.

## Introduction

Barnacles are a unique class of sessile arthropods that interest researchers for a variety of reasons: as hard foulants on seaborne fixtures and vessels [1–3]; for their ability to settle and develop on an impressive array of man-made and living objects [4–13]; and in an historical context due to their rich diversity, which has prompted Darwin and others to use them as a model for, among other things, the evolution of mating systems [8, 14–19]. In regard to the latter, Darwin provided detailed sketches of acorn barnacle anatomy along with descriptions ranging from barnacle metamorphosis, reproduction, cement production, and taxonomy [8, 14]. While larval and adult acorn barnacle anatomy have been studied and related to various aspects of their general biology [8, 20–23], certain aspects are poorly understood for a very practical reason: processes related to growth, cementing, and reproduction, particularly at the leading edge of the barnacle basis, are confined within a relatively small area and are obscured by parietal plates (side shells) and, in coronuloids and balanids, a calcareous base plate.

**Fig 1:**
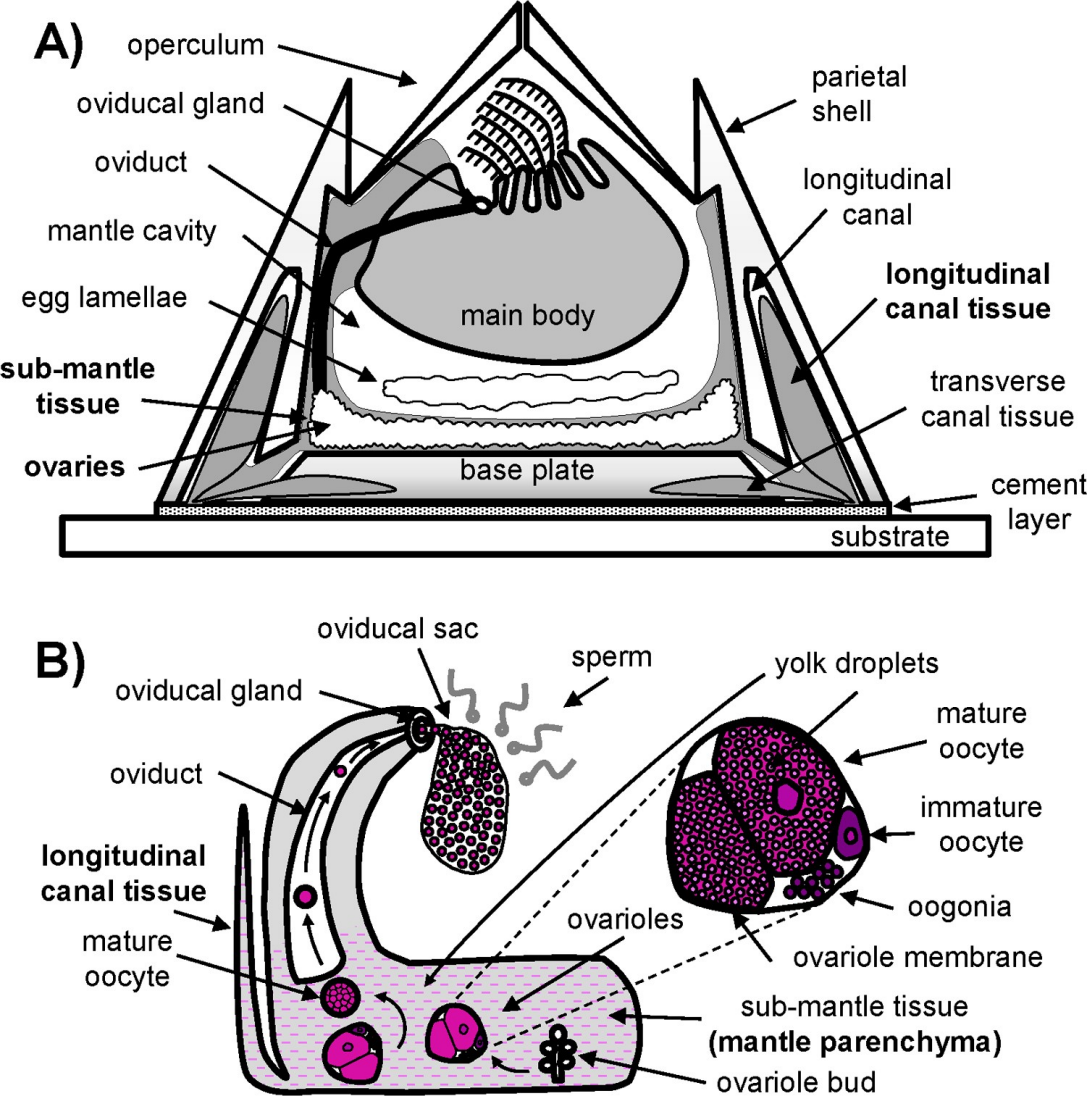
Generalized schematic of acorn barnacle and female reproductive tissue. A) Sagittal schematic highlighting the main body, the main components and showing the location of the LCT, and the sub-mantle region. B) Schematic identifying different structures of the female reproductive system and its relation to LCT.

Descriptions of the acorn barnacle main body have been available for over a century [8, 21, 22]. The main body is located beneath opercular plates and surrounded by a mantle (Fig 1A). Underneath the mantle cavity are features that can roughly be categorized as connective tissue, female reproductive tissue, muscle, and an open circulatory system consisting of interconnected capillaries that trace back to two principal canals originating in the central region of the barnacle and terminating as small (~10μm diameter) capillary ducts at the substrate interface in a radially distributed pattern. Several reports have detailed this sub-mantle region, mostly through fixed tissue sections made after dissection or decalcification of the shell [21, 24–26]. In particular, the female reproductive system, consisting of readily identifiable ovarian tissue (ovarioles) undergoing oogenesis, has been described [24, 26]. Germinal cells, or oogonia, within the ovarioles have been identified as the primary cells responsible for oocyte development [27, 28]. Although it has long been known that large, adult acorn barnacles are hermaphrodites, some details of their reproductive system, including essential components conserved among model hermaphrodites and dioecious arthropods, have not been clearly identified and are relatively unexplored. For instance, the ovarioles of invertebrates often consist of a nutrient-supplying tropharium or germarium where oogonia emerge and a vitellarium in which the oocytes develop and mature. As described in the histological analysis of Fyhn and Costlow, the distinction between the germarium and vitellarium in *Amphibalanus (= Balanus) amphitrite* is lacking [26]. (For the purposes of promoting continuity in the literature, we note the naming convention *Amphibalanus (= Balanus) amphitrite* since there was a period when the same species was labeled under the genus *Balanus*. See ref [29]). Both were suggested to be present within ovarioles: the germarium was described as being distributed throughout the ovariole and the vitellarium as consisting of oocytes with no apparent accessory cells. Hence, a complete understanding of the female reproductive system in acorn barnacles, specifically *A*. *amphitrite*, remains elusive. A greater understanding could ultimately provide crucial information on how sexually important structures develop and mature from the period of cyprid settlement to fully mature individuals. In addition to the germarium and vitellarium, other features are expected but have not yet been identified in the barnacle reproductive system, including: nurse cells which supply nutrients to early stage oocytes [26, 28]; follicle cells that provide yolk materials to enlarging oocytes [26]; and the accessory gland which is responsible for production of materials to package oocytes. Insight into the location of these components ultimately will broaden our understanding of barnacle growth and development.

Herein, we describe and analyze tissue located within the canals of the parietal plates of *A*. *amphitrite*. This region of the barnacle has been described previously with attention given to various aspects including the morphology as it relates to taxonomy [8, 30, 31], structural development and analysis [21, 25, 32], and the role of the epithelial layer surrounding the tissue of interest [33]. For example, the cells lining parietal plate canals (referred to as matrix-secreting cells) have been implicated in the initial formation of the longitudinal septa in *Balanus improvises* juveniles [25] and with biomineralization as barnacles expand their calcareous shells [33]. Though the shell architecture differs substantially between barnacle species [34–36], the vertical canals of *A*. *amphitrite* are located distinctly within the parietal plates and complement horizontal canals within the base plate.

For this study, we are interested in the tissue within the vertical canals, designated as longitudinal canal tissue or LCT, in *A*. *amphitrite*. Based on histological, proteomic, and lipid analyses, we propose one of many possible functions of LCT is related to the female reproductive system located in the mantle parenchyma of the barnacle, most likely providing precursor materials to developing oocytes in the ovarioles. We utilize a sub-mantle tissue transcriptome [37], which we translated and previously used to identify novel proteins in barnacle cement and confirm the presence of several enzymes at the substrate interface [38, 39]. Proteomic analysis of LCT and the sub-mantle tissue via mass spectrometry reveals a nearly 50% overlap of the protein profile with the greatest number of significant sequence matches to the glycolipoprotein vitellogenin. This complex is a dominant factor in vitellogenesis, which is the process of accumulation and deposition of nutrients in oocytes. Combined, these data suggest LCT in *A*. *amphitrite* is, at least in part, related to the female reproductive system. The findings promote the understanding of barnacle reproduction while also raising additional questions regarding the function of various features located at the leading edge and barnacle basis, where a combination of cementing, molting, and reproductive development processes are active.

## Materials and methods

### *A*. *amphitrite* husbandry

Sexually mature *A*. *amphitrite* barnacles were settled as cyprids on glass slides or silicone-coated glass panels and reared at Duke University Marine Laboratory (Beaufort, NC) [40, 41]. Adult barnacles shipped to the Naval Research Laboratory (Washington, DC) were maintained in an incubator operating at 23°C on a 12-hour day/night cycle in artificial seawater. Barnacles were fed *Artemia* spp. nauplii (Brine Shrimp Direct, Ogden, UT) three times a week. The artificial seawater was changed once a week and excess algal growth was removed via gentle brushing.

### Histological sectioning

Barnacles (base diameter ~ 1 cm) on two different substrates were used for sectioning. The first were settled on glass microscope slides. The second were removed from silicone panels and re-attached [41] to small pieces of overhead transparency film (3M Transparency Film PP2200, 99% solvent free). The latter were grown on the film for 6 weeks and served as a means to preserve basal tissue for sectioning, in particular sagittal sections spanning the height of the barnacle and through the film substrate. For fixation and decalcification, barnacles were fixed in 10% neutral buffered formalin (NBF) for at least 12 hours, then transferred to 25% formic acid (FA) solution buffered with sodium citrate overnight. The solution was changed, then the specimens rinsed and processed under vacuum filtration as per well-established formalin-fixed paraffin-embedding (FFPE) specifications. Processed barnacles were paraffin embedded and sectioned in either a sagittal or transverse orientation in 5–7 micron increments. Established protocols for hematoxylin and eosin (H&E) were used to stain every 5^th^ section with additional sections remaining embedded in paraffin for subsequent analysis.

### Section staining

The paraffin embedded histological sections were deparaffinized in absolute xylenes and immersed in 100%, 95% and 70% (v/v) ethanol in a stepwise manner for rehydration. Subsequently, the sections were incubated with select fluorophores in 1× PBS at 37 °C for 30 minutes: BODIPY FL (lipids, Thermo Fisher), SYPRO Ruby (protein, Thermo Fisher), and 4',6-diamidino-2-phenylindole (DAPI) (nuclear material, Thermo Fisher).

### Imaging & analysis

Sections stained with H&E were imaged on a Nikon Eclipse E600 Pol inverted microscope under various magnifications. Confocal fluorescent images of stained sections were collected on a Nikon A1R+ laser scanning confocal microscope and captured using standard detector settings for DAPI, fluorescein (to image BODIPY FL), and Cy3 (to image SYPRO Ruby).

Barnacles (average basal diameter 5–10 mm; height 3–5 mm) partially demineralized to expose LCT were prepared using Morse’s solution (22.5% formic acid + 10% sodium citrate). Unfixed shells were exposed for up to 1 hour, until the parietal plates dissolved, exposing the longitudinal canals. Shells from other barnacle samples were fixed in 4% formaldehyde for 24 hours. In these samples, some shell material remained and LCT was separated and dissected from the remaining soft tissue.

Images of intact LCT from partially demineralized barnacles were collected on a dissecting microscope or the Nikon confocal system utilizing the transmission detector. Freshly detached LCT freed from parietal shell canals was imaged under high magnification to quantify the size of abundant lipid-like droplets distributed throughout the LCT as well as for the presence of protein and cell nuclei. This was performed by staining the tissue with BODIPY FL, SYPRO Ruby, and DAPI and imaging using fluorescent confocal microscopy. Subsequent analysis of the droplet size distribution was performed using Nikon Elements imaging software.

### Proteomics

The mantle parenchyma, which includes the female reproductive tissue, and longitudinal canal tissue were collected from two sexually mature barnacles (basal diameter ~ 1 cm) grown on silicone substrates. Samples collected from each barnacle were processed independently via pressure cycling technology (PCT) [42]. After cleaning the outside shell, the operculum and main body were removed to allow access to the sub-mantle tissue which is attached to the inner surface of the parietal and base shells. As much sub-mantle tissue as possible was removed with the parietal shells intact and placed in a PCT MicroTube. Care was then taken to isolate LCT in the following manner: the base plate was carefully pulled away from the side shells, and all remaining soft tissue lining the inside of the parietal plates was removed. The tissue remaining after this separation was the isolated LCT. The cleaned parietal plates were dissolved by placing them in 22.5% FA and 10% sodium citrate solution between 30 to 60 minutes, until the shell material had completely dissolved and the longitudinal canals could be collected, rinsed, and placed into PCT MicroTubes tubes. This procedure minimized contamination prior to mass spectrometry analysis.

The LCT and sub-mantle tissue samples were homogenized using PCT in lysis buffer (30 µl/sample of 8M urea in 100 mM ammonium bicarbonate with protease inhibitors) in the Barocycler NEP 2320 (60 cycles of 20s at 45 kpsi and 10s at ambient pressure, RT). Cysteines were then reduced and modified by adding tris(2-carboxyethyl)phosphine (TCEP) to 10 mM and iodoacetic acid (IAA) to 40 mM in a total volume of 40 µl and incubating in the dark for 30 minutes [43]. Sequential digestion was performed by first adding 10 µl of 0.4 µg/µl of Trypsin/LysC Mix (Promega) and 5 µl n-propanol (Barocycler: 45 cycles of 50s at 20 kpsi and 10s at ambient pressure, 37°C) and then adding 85 µl 100 mM ammonium bicarbonate and 10 µl n-propanol (Barocycler: 90 cycles of 50s at 20 kpsi and 10s at ambient pressure, 37°C). Samples were then desalted with Strata-X 30mg/1ml columns (Phenomenex) and SpeedVac lyophilized and stored at -80°C.

Peptides were brought up in 100 µl of 0.1% FA in water and analyzed by liquid chromatography mass spectrometry/mass spectrometry (LC-MS/MS) with a Tempo-MDLC coupled to a TripleTOF 5600 mass spectrometer (AB Sciex, Foster City, CA). Samples were loaded onto and eluted from dual 3 µm 120 Å ChromXP C_18_CL RP Columns with a gradient from 80:5 to 5:80 0.1% FA in H_2_O:acetonitrile over 140 minutes. Tandem mass spectra were extracted and searched against the BarnALL database using Mascot as described in So et al. [38]. The BarnALL database was generated from translated cDNA sequences produced from RNA-seq experiments of the sub-mantle tissue [37] and also contains the 52 proteins identified in the shell [44]. Samples were analyzed assuming trypsin digestion with a peptide and MS/MS tolerance of 0.6 Da. Deamidation and oxidation were listed as variable modifications in the Mascot search parameters. Scaffold was then used to assess and verify peptide and protein assignments (peptide threshold > 95%, minimum number of peptides = 2, protein threshold > 99.0%; FDR for peptides = 0.42% and for proteins = 0.0% at these settings) as well as to perform quantitative analysis (ANOVA, p < 0.05, Benjamin-Hochberg multiple test correction).

Identified proteins were then used to compare the content of LCT and sub-mantle tissue collections, specifically using the top 50 proteins identified from LCT samples (based on assigned spectrum hits). A logarithmic comparison was used to emphasize the range of spectrum hits within the top 50 proteins. These data were used to create a heat map in R with the gplots package. The 20 proteins found only in LCT but in both replicates were also further examined. Combined, these 70 proteins were annotated using BLASTp and their Biological Process Go Terms were examined using Uniprot. The mass spectrometry proteomics data are publically available at the ProteomeXchange Consortium via the PRIDE partner repository with the dataset identifier PXD010743 (http://www.ebi.ac.uk/pride).

### Lipid analysis

For lipid analysis, barnacles were first cleaned under deionized water and the operculum, main body, and sub-mantle tissue were removed, leaving as little residual tissue attached to the parietal shells. Emptied shells were then treated for 3.5–4.5h in 0.25M HCl with gentle agitation until the LCT was clearly visible and was not covered by shell or membrane tissue. LCTs were manually plucked from the remnants of dissolved parietal shell, with care to avoid other non-LCT tissues, and then frozen at -80°C until extraction.

Lipid extraction was performed essentially according to the Folch method [45]. Pooled LCTs from 40–45 barnacles were homogenized in an all-glass homogenizer in 0.5 mL 2:1 chloroform:methanol until large particulates were no longer visible. The slurry was covered and allowed to rest for 15 min, then rehomogenized with an additional 0.5 mL chloroform:methanol. The solution was transferred to a separatory funnel and the homogenizer rinsed thrice with 1 mL chloroform:methanol for a total of 4 mL LCT solution. Saline (0.8 mL) was added and the separatory funnel mixed for approx. 10 min before separation of aqueous and organic phases. The organic phase was dried down and stored at -20ºC under N_2_ until analysis via Thin Layer Chromatography (TLC) on silica G60 (Merck) using 75:25:2.5 chloroform:methanol:water. Iodine vapor was used for initial localization of spots on TLCs before subsequent staining. Dragendorff reagent, ninhydrin, and Molybdenum Blue reagent [46] were purchased from Sigma-Aldrich and were used as-is for detection of phospholipids, primary amines, and substituted amines, respectively. A 2% solution of 2,3-dichloro-5,6-dicyano-1,4-benzoquinone in toluene (DDQ reagent) and Dreywood’s anthrone reagent were used as described to detect phenolic and carbohydrate components of LCT extracts, respectively [47, 48]. Staining of TLCs with resorcinol-trichloroacetic acid (sugars), α-naphthol-sulfuric acid (carbohydrates), Ehrlich’s reagent (primary amines), phosphoric acid (sterols), perchloric acid (sterols, bile acids) were performed essentially as described by Merck [49].

## Results

### LCT location and general description

**Fig 2:**
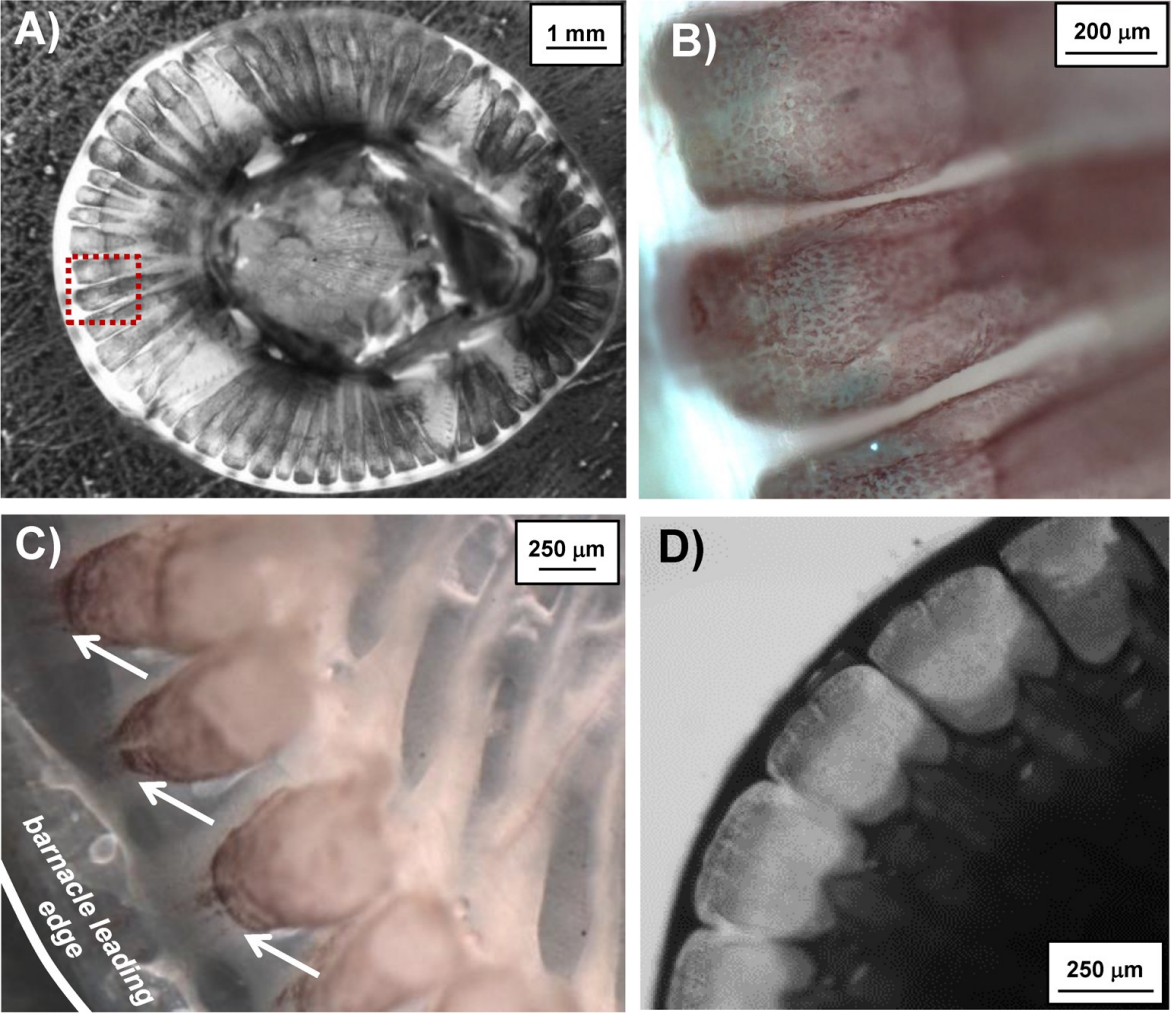
Brightfield images of partially demineralized *A*. *amphitrite* and exposed LCT. A) Top view of partially demineralized *A*. *amphitrite* with LCT exposed at the periphery. Dashed red box represents area under higher magnification in panel B), showing greater detail of the exposed LCT including the pink hue arising from the speckled epithelial layer. C) Image of exposed LCT showing their termination into channel-like projections toward the barnacle leading edge. D) Top-down view of partially demineralized *A*. *amphitrite* highlighting the longitudinal cavities containing the tissue of interest.

A general scheme of an acorn barnacle in the sagittal plane is shown in Fig 1A highlighting various major tissue components. A zoomed schematic identifying components of the female reproductive system is given in Fig 1B. The LCT is embedded within the calcium carbonate parietal plates (Fig 1A) and was exposed by partial decalcification of the shell in adult, *i*.*e*. sexually mature *A*. *amphitrite*. Fig 2A shows the LCT in a barnacle appearing in clusters that correspond to the size of the different parietal plates. In general, the tissue within the canals of *A*. *amphitrite* matches the dimensions of the canals, which have biomineralized around the tissue. Further, LCT is encapsulated with a sac-like membrane; intact sacs of LCT can be gently removed from decalcified side shell plates. Piercing this membrane results in the contents diffusing into the surrounding milieu. The outer surface, which previously has been identified as epidermal layer [33], has a pink hue and a speckled appearance (Fig 2B). When decalcification is performed with EDTA over the course of several hours, the reddish color rapidly fades away suggesting the presence of metalloproteins [50]. When exposed LCT is viewed in transmission (Fig 2C), the lower portion of the tissue appears to terminate as finger-like projections toward the leading edge of the barnacle-substrate interface. In younger adult barnacles, where the calcified parietal shell was thin enough to allow limited brightfield transmission, particulates were observed moving through the LCT projections in an undirected manner resembling Brownian motion. Fig 2D shows a top-down view of the parietal shell canals of a young, intact barnacle, highlighting the space in which LCT is confined. To gain further insight into the composition, LCT sacs were extracted and stained against major classes of biomacromolecules.

### Biomacromolecular staining profile

**Fig 3:**
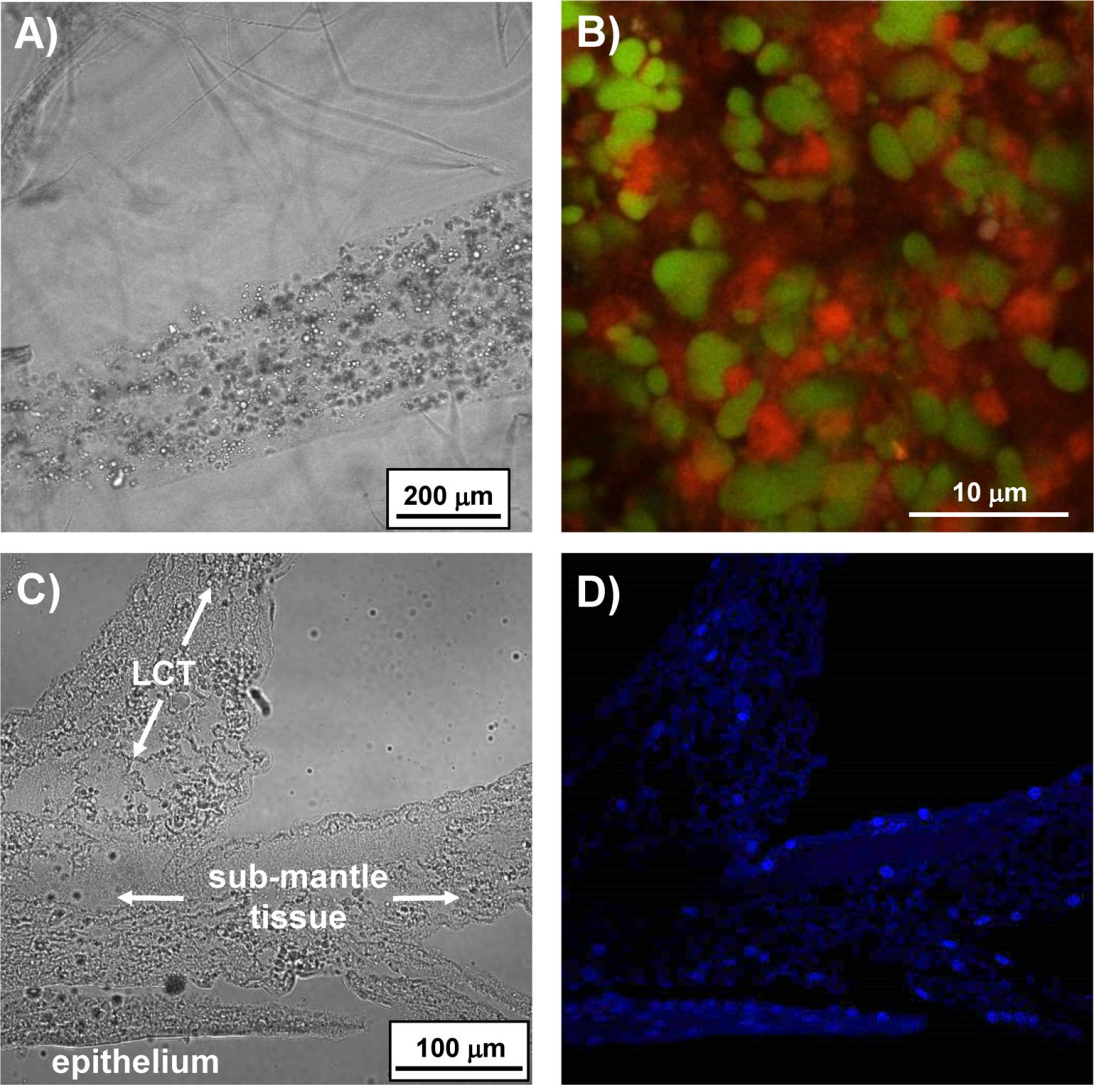
Images of exposed LCT. A) Brightfield image under lower magnification showing the interior texture. B) Higher magnification image of LCT stained with Bodipy FL (green, lipophilic) and Sypro Ruby (red, protein). C) Brightfield and D) confocal image of a sagittal section of LCT and submantle tissue stained with DAPI showing the presence of nuclei throughout. An epithelial layer is also present along the bottom edge.

Freshly exposed and extracted LCT stained with fluorescent dyes against lipid (BODIPY FL), protein (SYPRO Ruby), and nucleotides (DAPI) revealed all three biomacromolecular classes to be present (Fig 3). BODIPY FL had a distinct profile in that it only stained small spherical structures within the LCT. Confocal microscopy confirmed staining through the entire volume of the spheroids and not just the lipophilic membrane as one may expect with a vesicular structure. Given the relative abundance of the droplets in LCT, the size distribution was quantified from confocal images of LCT stained with BODIPY FL. The images were captured from several adult barnacles (n = 5) within 2 hours of being sacrificed and exposed to HCl treatment; this was critical since it was observed that droplets began to coalesce within hours. Analysis of hundreds of particles revealed the average diameter to be 1.9 ±0.7 µm with a range spanning 0.5 to 5 μm.

Protein staining with SYPRO Ruby revealed the majority of other tissue within LCT to be a proteinaceous matrix surrounding the lipophilic spheroids. As shown in Fig 3B, dual staining against protein and lipid confirmed the droplets were lipophilic, with material surrounding the droplets staining positive for protein. Separate DAPI staining of LCT revealed the presence of nuclei (Fig 3C and 3D) within LCT, indicating the presence of cells. The nuclear distribution was relatively sparse given the volume of LCT within each sac. It is noteworthy that confocal microscopy showed nuclei were inside the LCT sacs and distinct from the epidermal layer present at the surface of freshly exposed LCT (see Fig 2B).

### H & E stained histological sections

**Fig 4:**
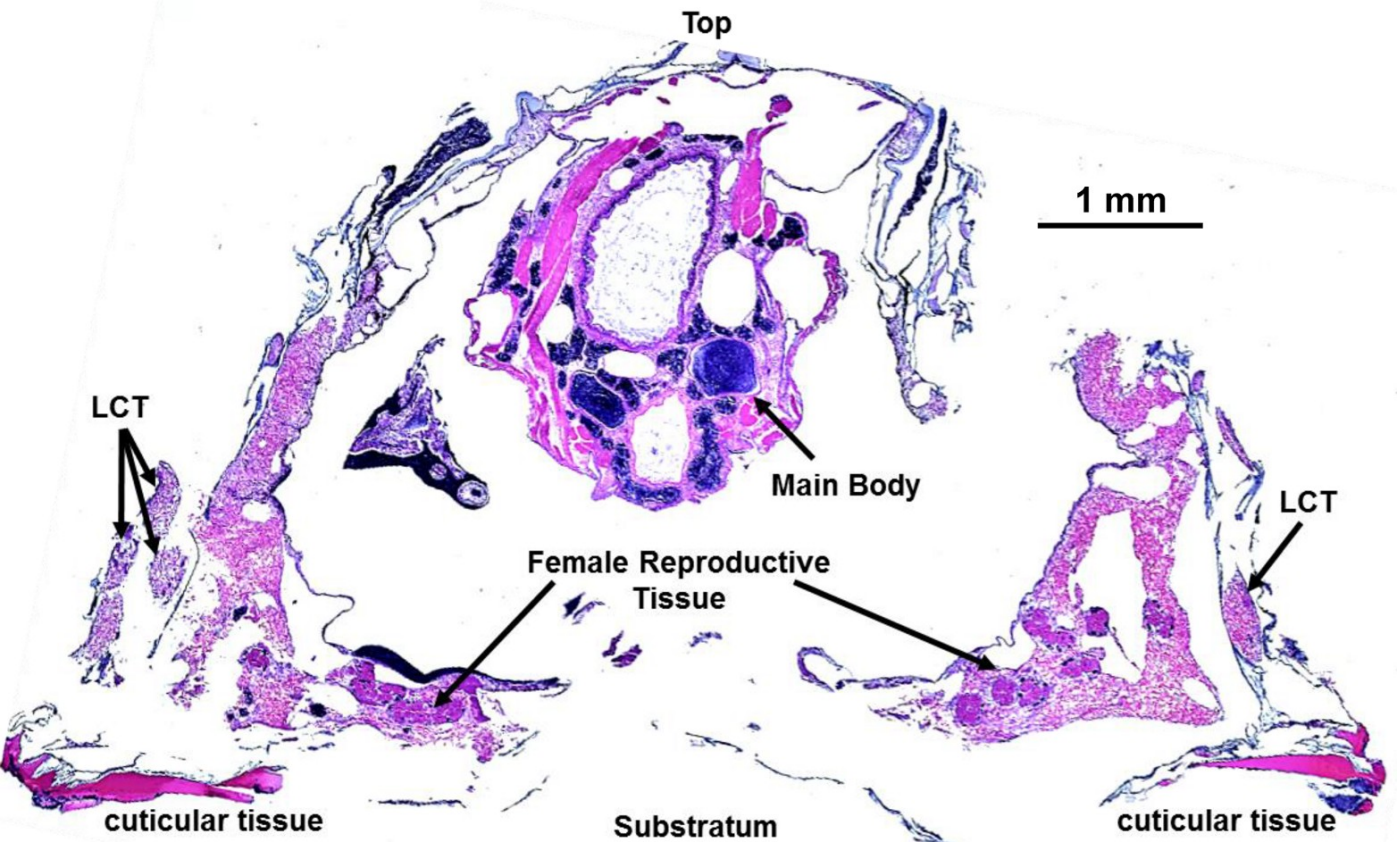
H & E stained sagittal section of *A*. *amphitrite*. Four prominent components are labeled: portions of the LCT, the main body, female reproductive tissue, and underlying cuticular tissue.

**Fig 5:**
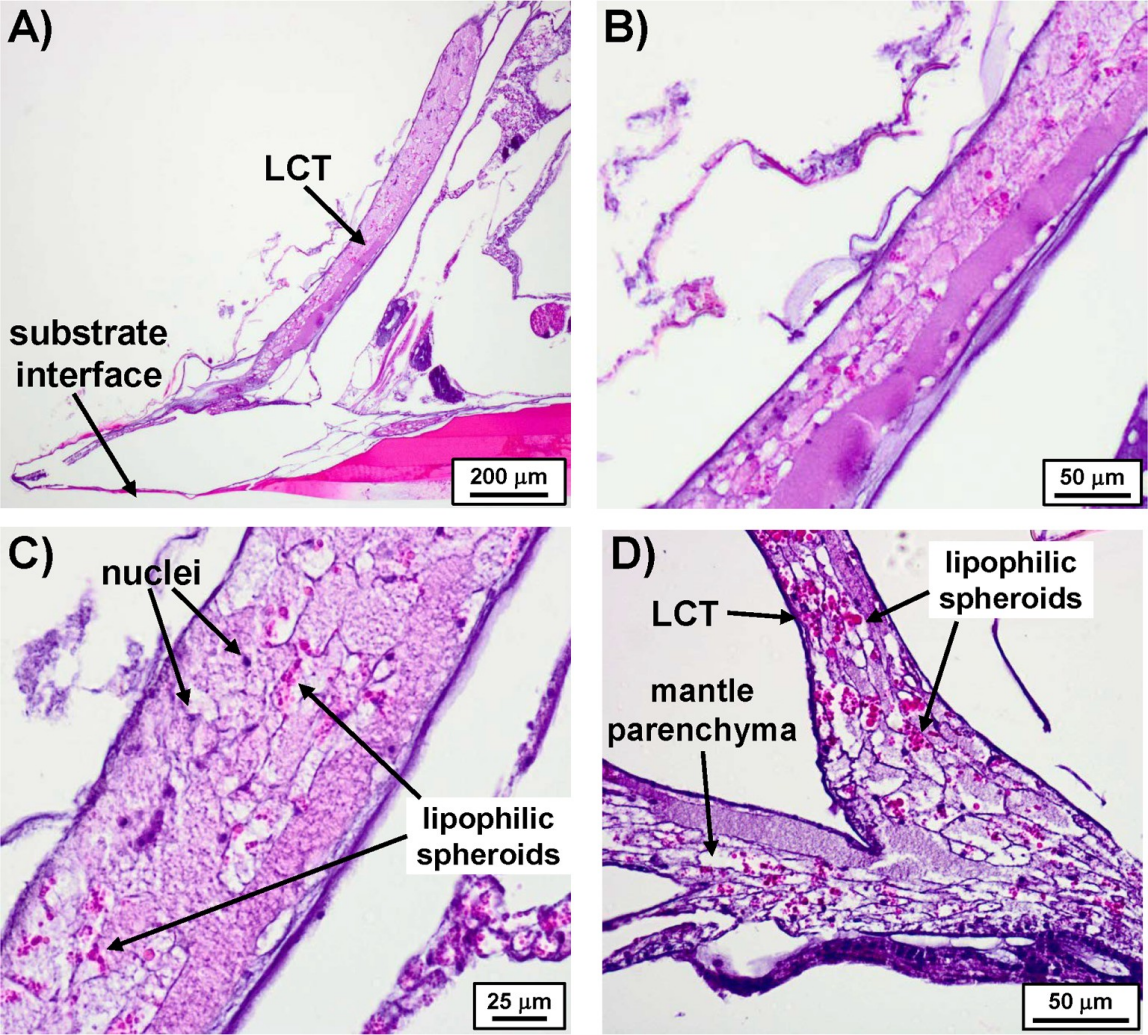
Sagittal sections of LCT stained with H&E. A) Entire view of LCT in relation to the edge of *A*. *amphitrite*. B) Magnified image of A) showing the various textures and staining profile. C) Another zoomed image of A) highlighting the presence of nuclei and clusters of lipophilic spheroids. D) Sagittal section showing connection between the LCT and the mantle parenchyma.

**Fig 6:**
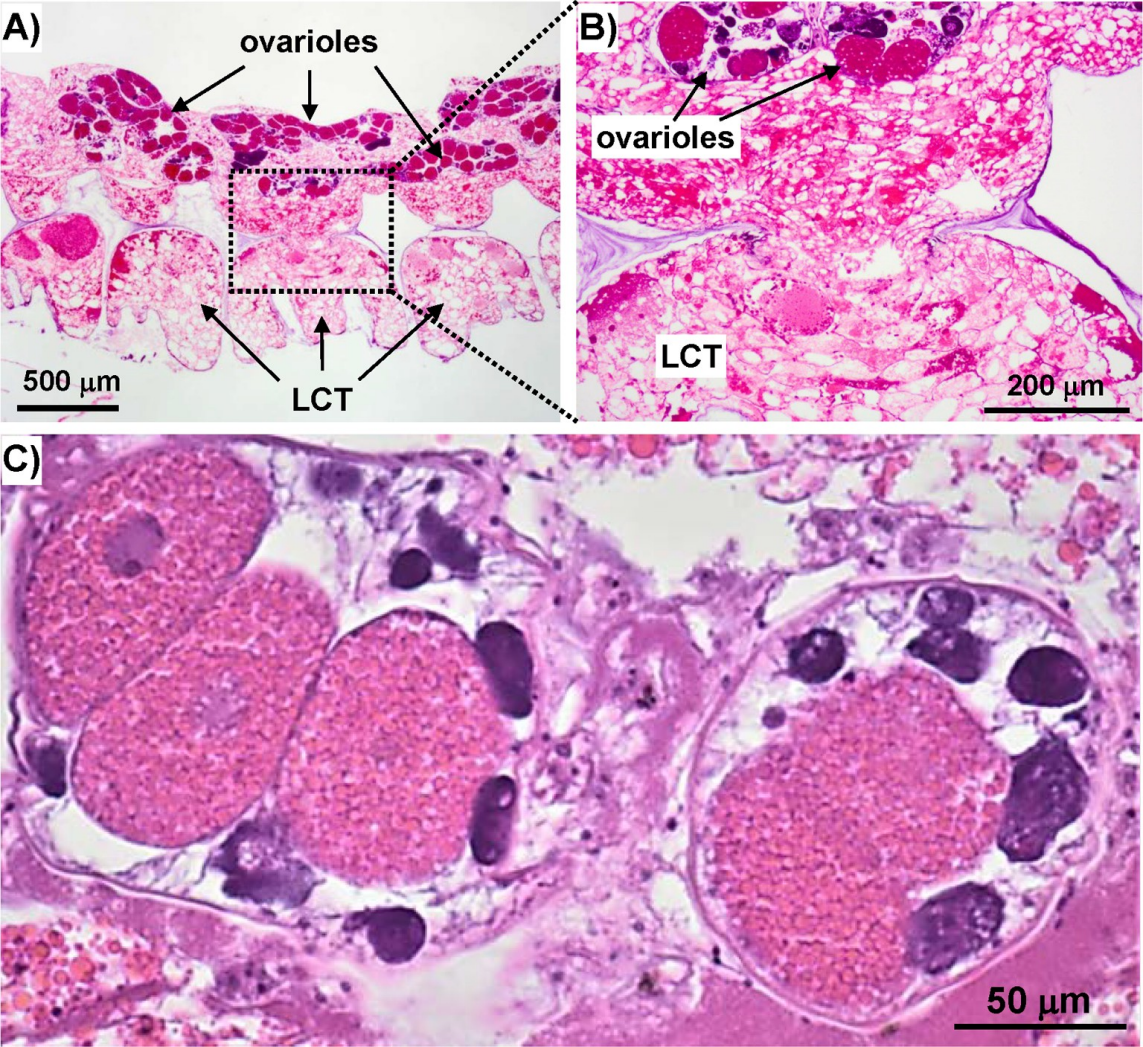
H&E stained transverse *A*. *amphitrite* sections. A) Low magnification image of LCT and sub-mantle tissue containing the ovaria. B) Higher magnification image of A) highlighting continuity between LCT and sub-mantle tissue. C) Image of two ovaria showing oogenesis at different developmental stages.

To gain further insight into the composition of LCT and its relation to other tissue, transverse and sagittal sections of fixed, decalcified barnacles stained with hematoxylin and eosin (H&E) were imaged. Fig 4 shows a sagittal section of an entire barnacle highlighting some of the major soft components including the main body, female reproductive tissue in the sub-mantle cavity, cuticular tissue at the basis, and LCT at the periphery. (A complementary transverse section of *A*. *amphitrite* is provided in the Supporting Information, S1 Fig) Despite some of the spatial distortion in the sections inherent from sample preparation and the dehydration process, we draw attention to the proximity of LCT with respect to tissue within sub-mantle cavity that lines the interior of the basal margin of the parietal plates and above the base plate. Among the various tissue types within this region, the distribution of readily identifiable ovarioles (and its proximity to LCT) is noted. The general morphology and staining pattern of the basic stain hematoxylin (blue/violet, commonly staining DNA) and acidophilic eosin (red/pink, commonly staining cytoplasmic protein) in LCT is highlighted in sagittal and transverse sections in Figs 5 and 6, respectively. Observing histological sections from several barnacles, both views confirm the existence of the hematoxylin-stained, sac-like membrane surrounding the LCT with an epidermal layer. The interior portion of LCT is compartmented with a range of textures and various staining profiles. As shown in Fig 5A–5C, portions of the tissue have a smooth texture and stain pink/violet. Other regions have a similar hue though the texture is roughened, looking more like a segmented, disorganized matrix.

The H&E stained sections reveal two common features distributed throughout LCT. First, hematoxylin-stained spots resembling nuclei were present within LCT and had a sparse distribution (Fig 5C), correlating directly with observations using DAPI with confocal microscopy. Second, eosin-stained spheroids were distributed throughout the tissue, often in concentrated clusters (Fig 5C and 5D). These correlate with the lipophilic spheroids observed with BOPIDY FL using confocal microscopy. The persistence of these structures after sectioning and staining is of particular note since, in general, pure lipidic tissue will not remain intact after the treatment of xylene that precedes H&E staining.

The other notable feature of LCT sections is its connection with the sub-mantle tissue (*i*.*e*. mantle parenchyma), which shows the same texture with an added abundant distribution of ovarioles, which were absent in LCT. By our own definition LCT is largely confined in the parietal shell, but sagittal sections of the barnacle clearly show the base region of LCT is continuous with the mantle parenchyma surrounding the ovaries. The textures of the two regions are morphologically indistinguishable and possess the same staining profile (Fig 5D compared to Fig 6A and 6B). Fig 6A and 6B also shows the relative proximity of LCT to ovarioles, *i*.*e*. within a few hundred microns of one another. As noted, a primary constituent found in the sub-mantle tissue is the female reproductive system, with readily identifiable ovarioles broadly distributed at the periphery of the mantle parenchyma (Figs 4 and 6A and S1) and at various stages of egg development (Fig 6C). One common feature in LCT, the sub-mantle tissue, and the ovarioles is the eosin-stained spheroids, which are most concentrated within the mature stages of oocyte development and correlate with the absence of nuclear staining. Given the proximity of LCT to the mantle parenchyma containing ovarioles, a proteomic approach was employed to compare these two tissue types and gain insight into its potential function(s).

### Proteomic comparison of LCT and sub-mantle tissue

**Fig 7:**
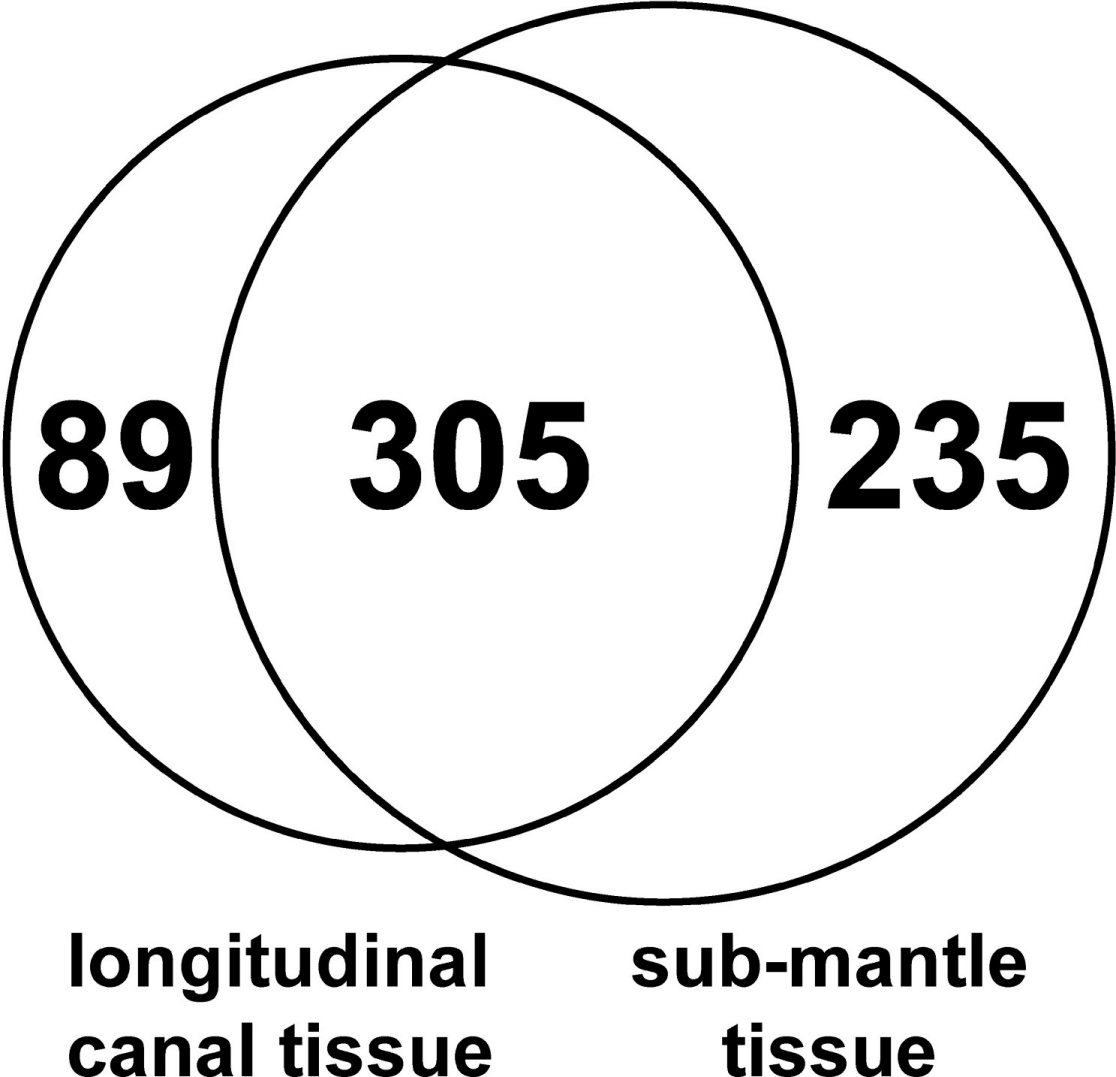
Venn diagram providing an overview of the protein profile of LCT and sub-mantle tissue. Out of a total of 629 identified proteins, 48% (305) were identified in both tissues.

The proteome of tissue gathered from the sub-mantle region of barnacles was compared to the protein profile of LCT collected from decalcified parietal shells using mass spectrometry. As the LCT and the sub-mantle tissue are in close proximity, great care was taken to ensure there was minimal cross tissue contamination (see Methods). A total of 629 proteins were identified (see Supporting Information S1 Table) and of those 305 (48%) were shared between the two tissues (Fig 7). Overall, more proteins (235) were found to be unique to the sub-mantle tissue. This result is unsurprising as the database used for protein identification was generated from transcripts found in the sub-mantle tissue [37], which likely biases protein identification in favor of this tissue. Of the 89 proteins unique to the LCT, 20 were identified in both replicates (S1 Table). Of this subset, 9 are predicted to be related to metabolism, 6 to cell morphology, 3 had no or low homology to known proteins, and 2 to the immune response.

**Fig 8:**
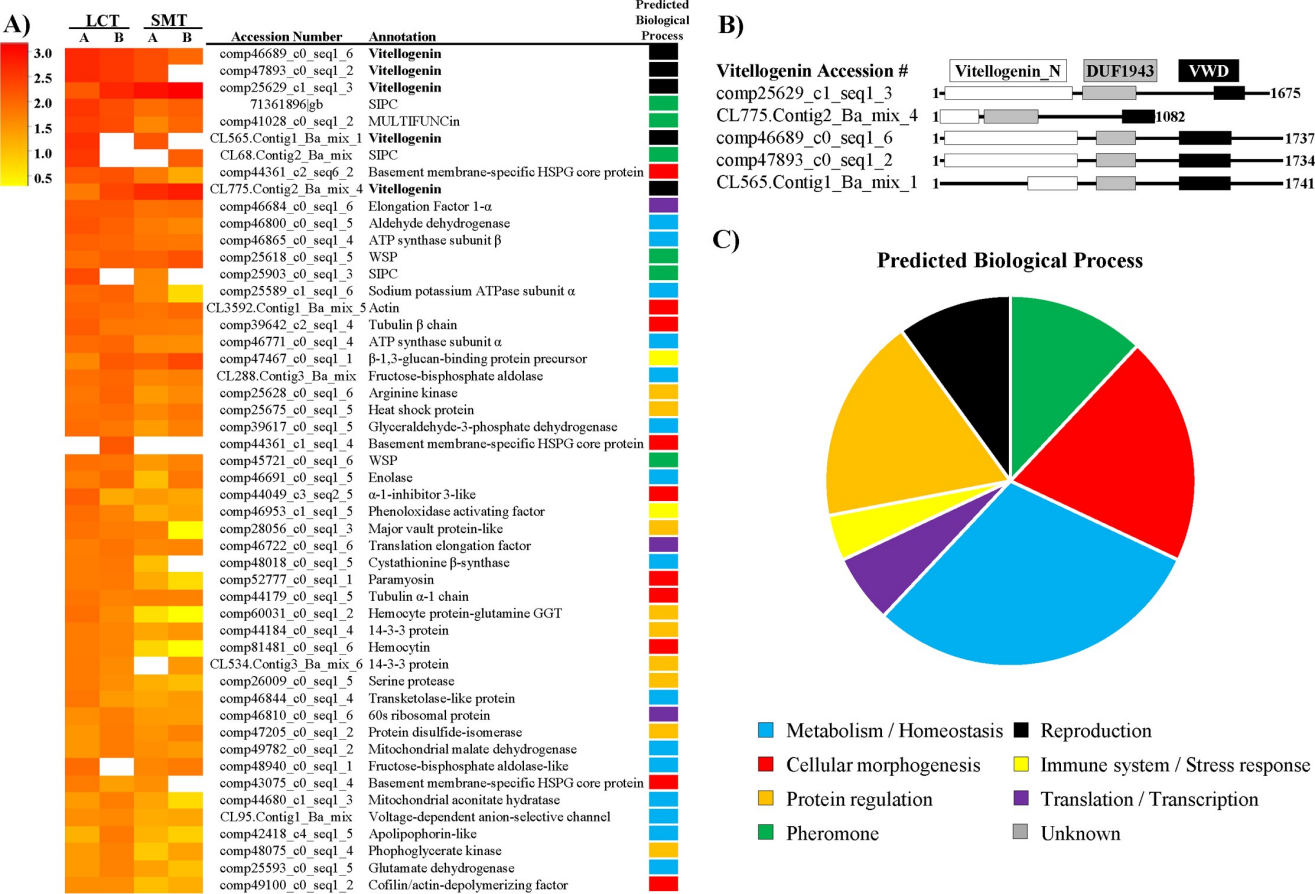
Overview of top 50 proteins identified within LCT. A) Heat map of logarithmically transformed data. Spectra assigned to vitellogenin are in bold. Data are sorted by the combined average of the LCT A and B values. B) Position of conserved domains present in *A*. *amphitrite* vitellogenin proteins: Vitellogenin_N superfamily, DUF1943 (Domain of Unknown Function), and VWD (von Willebrand Domain) superfamily. C) Predicted general biological processes for the proteins in A). Abbreviations: LCT: longitudinal canal tissue; SMT: sub-mantle tissue; SIPC: settlement inducing protein complex; HSPG: heparan sulfate proteoglycan; ATP: adenosine triphosphate; WSP: waterborne settlement pheromone; GGT: gamma-glutamyltransferase.

The heat map in Fig 8A displays the top 50 proteins identified in LCT (based on the number of assigned spectra) compared to those in the sub-mantle tissue. The BlastP results for these proteins are included in S2 Table and the resulting annotation and biological process are described in S3 Table. Based on the number of spectra, five out of the top ten most abundant proteins were annotated as vitellogenin, a yolk protein precursor important primarily for reproduction. The presence of three conserved domains (Vitellogenin_N superfamily, Domain of Unknown Function [DUF]1943, and von Willebrand factor domain [VWD] superfamily) further strengthens the categorization of these proteins as vitellogenin [51] (Fig 8B). Several pheromones (settlement inducing protein complex (SIPC), MULTIFUNCin, and waterborne settlement pheromone (WSP)) were also present in high abundance. In addition to vitellogenin and pheromones, the other LCT proteins listed in Fig 8A fall into a wide range of predicted biological processes and functions: metabolism, cellular morphogenesis, protein regulation, immunity, and translation/transcription (Fig 8C). The metabolic category contains the most proteins, and the combined number of proteins involved with metabolism, translation/transcription, and protein regulation account for ~54% of the biological processes, indicating LCT is a metabolically active tissue. Also of note in the top 50 proteins in LCT are β-1,3-glucan-binding protein precursor and phenoloxidase activating factor (associated with immunity), and several enzymes with a range of functions. The significant overlap between the protein profiles from the two tissue types and the abundant presence of vitellogenin supports the histological analysis that LCT is associated with a female reproductive function.

### LCT lipid analysis

In addition to the protein profile, the organic, lipid-containing phase of LCT extracts was subjected to TLC to determine overall sample complexity and obtain a better understanding of the major lipidic components and biochemical moieties associated with them. At least six components migrated separately on TLC plates, with at least two components positive for the presence of phospholipid via molybdenum blue staining [52]. No staining of TLC plates was observed with ninhydrin, indicating that primary amines were absent. Positive staining with Dragendorff reagent indicated the presence of substituted amines or phenolics. The potential for phenolic content was further supported by positive reactions with DDQ and Folin-Ciocalteu reagents, although the latter reagent has broad cross-reactivity with other chemical moieties. The presence of sterols was suggested by colorimetric reactions with both perchloric and phosphoric acids; a blue fluorescent product formed with the latter.

The presence of a carbohydrate component in LCT organic extracts was ascertained through formation of a colored product with α-naphthol treatment. However, no color formation was observed with the ketose-specific stain, urea-HCl [53], but faintly colored spots were formed upon treatment with resorcinol (light pink) or anthrone-sulfuric acid (light brown/tan), suggesting the presence of sialic acid and/or other carbohydrate moieties.

## Discussion

The understanding of barnacle physiology has continued to broaden since the publication of monographs on the subject by Darwin [8, 14], yet critical aspects remain elusive. In acorn barnacles, this is mainly due to adults being confined within a calcareous shell, thus rendering dissection and microscopy a challenge. The primary alternative is observing the soft tissue exposed by a demineralizing agent, followed by histological sectioning. In the current study, dissection and sectioning have been employed to understand the role of tissue in the longitudinal canals of *A*. *amphitrite*.

In an intact barnacle, the vast majority of LCT is confined within the canals of the parietal plates with only the lowest portion extending near the leading edge of the barnacle basis. Several other notable features observed in both intact and sectioned LCT are summarized: *i)* the tissue is encased in a sac-like membrane and surrounded by an epithelial layer; *ii)* nuclei are present throughout LCT (as confirmed by the staining profile of both hematoxylin and DAPI); *iii)* transverse and sagittal sections reveal continuity between LCT and the mantle parenchyma–these two tissues are located in separate regions of the barnacle, yet are physically connected to one another at the LCT basis and show a high morphological similarity; and *iv)* micron-sized spheroids are distributed in much of the LCT as well as throughout the mantle parenchyma, which itself contains ovarioles with readily identifiable stages of oocyte development. These observations alone suggest LCT is related to the mantle parenchyma, which includes the female reproductive system of *A*. *amphitrite*.

As a backdrop, hermaphrodite arthropods such as *A*. *amphitrite* possess both male and female reproductive systems located in different regions of the body. The female reproductive system lies in a compacted tissue, *i*.*e*. the mantle parenchyma, extending along the internal perimeter of the mantle cavity and the parietal plates. The interior of the mantle parenchyma is adjacent to the rostral scutal depressor, which is opposite to the location of the penis. In the sub-mantle tissue, numerous connecting oocyte clusters develop in units designated as ovarioles, which are distributed densely over an area between the lateral scutal depressor and the rostral scutal depressor. This distributed system in the barnacle is expected to share several common features with the female reproductive system of other arthropods. For example, a typical arthropod ovariole consists of a germarium and a vitellarium. The former features nurse cells that support oogonia while the latter contains developing oocytes that uptake vitellogenin from surrounding follicle cells. Arthropod ovarioles are categorized into two types based on the presence of nurse cells: panoistic (nurse cells absent) and meroistic (nurse cells present). Oocytes grow autonomously in panoistic ovarioles whereas oogenesis is supported by nurse cells in meroistic ovarioles. Previously, neither nurse cells nor follicle cells have been positively identified in barnacles [26] and Walker suggested that oogenesis in cirripedes does not involve nurse cells [27]. This would lend support to barnacles having panoistic ovarioles.

Ultrastructural observations in this work suggest that, unlike dioecious arthropods with ovarioles organized into compact bundles and each ovariole containing a vitellarium with a well-defined boundary, *A*. *amphitrite* ovarioles have a pooled vitellarium with a direct connection to the longitudinal canal tissue. To support this line of thinking, LCT and sub-mantle tissue were collected for proteomic comparison via mass spectrometry. Using our in-house transcript library [37], 629 proteins were identified and 305 were found in both tissues. Of these common proteins, the three most abundant proteins were annotated as vitellogenin (Fig 8), a well-known yolk protein precursor involved in supplying nutrients to developing oocytes [54, 55]. Since this lipoprotein is generally known to be produced outside of the ovarioles and taken up by maturing oocytes as part of nutrient accumulation, we speculate that its presence in LCT relates this tissue to the female reproductive system in *A*. *amphitrite*. Further, it is possible that the lipidic spheroids in LCT, the sub-mantle tissue, and maturing oocytes are all of the same type, functioning as yolk granules. As noted in the Results, the composition of these spheroids is more than a simple lipid fraction as they persist following a xylene wash prior to H&E staining. For insight into the classes of lipid present in this tissue, TLC analysis of the lipid fraction collected from the organic phase was performed and the presence of phospholipid, sterols, and substituted amines or phenolics were indicated. These findings are generally in line with the findings of Dawson and Barnes in their lipid analysis of the egg development of *B*. *(= Semibalanus) balanoides* and *B*. *balanus*, where phospholipid and triglyceride were major components, with smaller amounts of sterols, free fatty acids, lipochromes, and other non-polar lipids also identified [56]. As with all arthropods, lipids serve a critical role as an energy reserve for the development of barnacle eggs, as highlighted in the overview of barnacle lipids and lipid synthesis by Holland [57]. While their role in egg development is established, it is noteworthy that lipids also appear to have an expanding role in barnacle cyprid settlement and the preparation and expansion of their adhesive interface [58, 59].

The results of our study indicate LCT has a role in the female reproductive system. The abundance of vitellogenin supports this claim, and is also indirectly supported a large number of proteins involved with general metabolism that were identified in both the LCT and the sub-mantle tissue. The protein profile shows LCT is an active tissue (and not vestigial) producing proteins necessary for reproduction, but also a variety of other functions. The strong presence of pheromones in LCT could aid in intraspecies chemical communication since proteins that function as settlement pheromones (settlement inducing protein complex (SIPC) [60], MULTIFUNCin [61], and waterborne settlement pheromone (WSP) [62]) were identified in relative abundance. There is also a potential role of LCT related to immunity based on some proteins identified via mass spectrometry. These include proteins involved in the prophenoloxidase activating system, which is an important innate immune response for invertebrates [63], C-type lectins [64], and reactive species scavengers like superoxide dismutase and peroxidase. Interestingly, oxygen radicals and peroxides have been implicated in barnacle adhesive formation at the substrate interface [39, 59, 65], though we note that a direct link between LCT and activity at the substrate interface has not yet been determined.

Though not as prominent, the proteomic data suggest that either the LCT (or the surrounding epithelial layer) is involved in biomineralization as several identified enzymes (carbonic anhydrase-, serine protease-, and peroxidase-like proteins) are likely contributors to shell hardening [44]. The in-depth examination of shell formation by Costlow in *B*. *improvises* provides further insight into the potential role of longitudinal canals [25]. While the *function* of LCT was not specifically addressed, Costlow noted the upper portion of longitudinal canals contained segmented tissue and the lower region (closest to the basis) was filled with what he termed “extensions of the mantle.” Though a different species, his sketches of the extended mantle directly correspond to our observation of the LCT being connected to, and morphologically indistinct from what we term the mantle parenchyma, which surrounds and supports ovarioles at the barnacle basis. The consistency of these observations in different species support the notion that LCT may be a common feature among several barnacle species and possesses similar function. We note that Costlow also raised questions as to the function of the transverse septa of the longitudinal canals themselves as being used for mechanical support or possibly vestigial structures. X-ray computed tomography provides evidence of the former [32].

In regard to reproduction, general observations of LCT and barnacle age also provide support for its role in reproduction. As noted by Costlow, shell development, specifically the formation of longitudinal septa, is initiated at day 4 of cyprid settlement and appears to be largely directed by matrix-secreting (epithelial) cells [25]. During this period, the barnacles are sexually immature so there is no need for LCT to be fully developed and functioning. After a few weeks the barnacle transitions to sexual maturity, which corresponds with fully formed longitudinal septa and the appearance of LCT. Since supporting tissue such as the germarium and vitellarium would be required for proper function of the ovarioles and oocyte maturation, it follows that LCT would also need to be functional. On the other end of the spectrum, collection of longitudinal canal tissue was much more difficult in older and larger barnacles where female reproductive tissue was not as abundant. In this case, the singular sac-like LCT units that were relatively easy to extract from younger barnacles were fragile, less intact, and had a degraded morphology. It is also possible that LCT may synchronize with the barnacle molt cycle. In our limited observation, we saw no evidence of this, though it was also outside of the primary scope of this effort.

## Conclusion

Examination of LCT in *A*. *amphitrite* with a combination of proteomics and histological analysis suggests a functional relationship to the female reproductive system. The data suggest LCT is likely to have several roles, though a few key indicators suggest this tissue may be responsible, in part, for reproduction-related nutrient production and storage. These include the existence of lipidic spheroids in LCT, the presence of vitellogenin, and the direct connection of LCT to the mantle parenchyma, which is morphologically indistinct and includes the ovarioles with oocytes in various stages of maturation. While our understanding of acorn barnacle reproductive physiology, specifically that of *A*. *amphitrite*, remains incomplete and there appear to be unique features, it is expected from an evolutionary perspective that we can continue to draw on the features common to other arthropods to expand our knowledge of these sessile marine organisms. Future efforts will focus on histological examination of other critical features located at the leading growth edge of the acorn barnacle basis.

## Supporting information

S1 Fig Composite images of histological section of *A*. *amphitrite* along transverse plane. Section is taken from the lower portion of the barnacle and highlights the presence of various features including: the lower parts of main body and testes, the distribution of ovarioles, LCT, and cuticular tissue.
(PDF)

S1 Table Excel file listing the 629 proteins identified in the sub-mantle tissue and LCT samples. (XLSX)

S2 Table Excel file listing the 20 proteins unique to LCT and identified in both replicates; BlastP results for the top 50 identified proteins in LCT. (XLSX)

S3 Table Excel file with the annotation and biological process of the top 50 proteins identified in LCT. (XLSX)
