# Supplementary material for: Characterization of longitudinal canal tissue in the acorn barnacle Amphibalanus amphitrite
Source: PLoS One. 2018 Dec 10;13(12):e0208352. doi: 10.1371/journal.pone.0208352 (PMC6287898; doi:10.1371/journal.pone.0208352)
Supplement: S1 Fig — Section is taken from the lower portion of the barnacle and highlights the presence of various features including: the lower parts of main body and testes, the distribution of ovarioles, LCT, and cuticular tissue. (PDF) [file pone.0208352.s001.pdf]

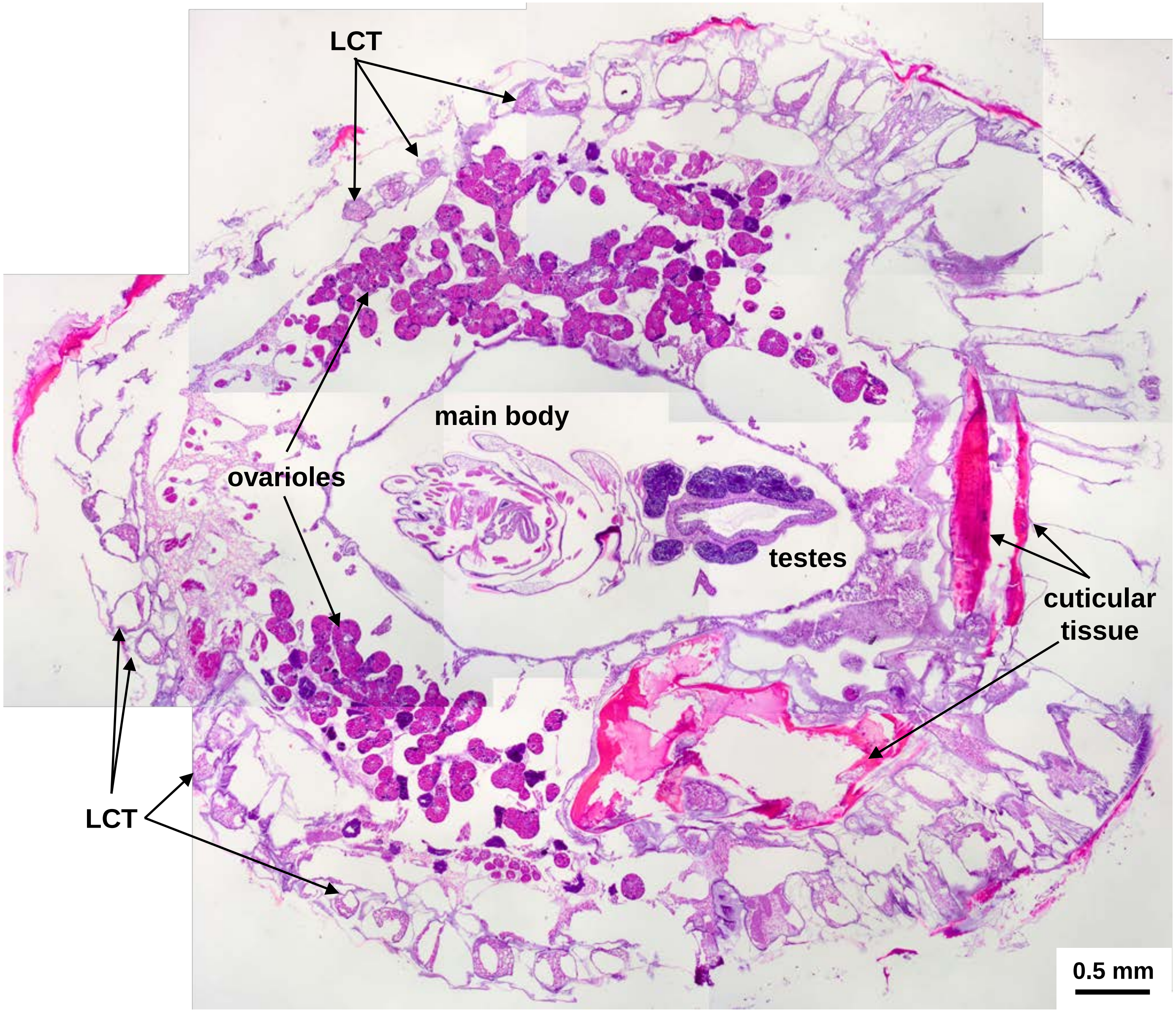

S1 Fig. Composite images of histological section of *A. amphitrite* along transverse plane. Section is taken from the lower portion of the barnacle and highlights the presence of various features including: the lower parts of main body and testes, the distribution of ovarioles, LCT, and cuticular tissue.
